# Case report: Co-occurring obsessive-compulsive disorder and affective psychotic disorder complicated by catatonia in an adolescent female patient

**DOI:** 10.3389/fpsyt.2023.1225827

**Published:** 2023-10-12

**Authors:** Wilson To, Crystal Yung, Tyler Voss-Hamrick, Brenner Meacham, Colin Freedman

**Affiliations:** ^1^Psychiatry Program, Sunrise Health GME Consortium, Southern Hills Hospital & Medical Center, Las Vegas, NV, United States; ^2^Touro University Nevada College of Osteopathic Medicine, Las Vegas, NV, United States

**Keywords:** catatonia, child and adolescent psychiatry, obsessive-compulsive disorder, case report, pediatric catatonia, psychosis, affective psychotic disorders

## Abstract

We present the case of a 16-year-old female patient who experienced the loss of her mother to suicide, leading to post-traumatic stress disorder and prominent mood symptoms. She developed catatonic features during her inpatient psychiatric hospitalization following her own suicide attempt. Over her hospital course, she began to demonstrate signs of co-occurring obsessive-compulsive disorder (OCD) and affective psychotic disorder obfuscated by the severity of her catatonia. After initial workup including neurologic evaluation, laboratory tests, imaging (EEG, MRI), the patient was stabilized on a combination of benzodiazepines, antipsychotics, mood stabilizers, and selective serotonin reuptake inhibitors. The diagnostic challenges of disambiguating multiple concurrent diagnoses in the presence of a syndrome with unclear pathophysiology are discussed. Recommendations are made to thoroughly evaluate thought content during periods of catatonic remission to guide diagnosis and treatment.

## 1. Introduction

Catatonia is a syndrome characterized by psychomotor disturbances that can range from vegetative immobility to frank agitation. By Diagnostic and Statistical Manual of Mental Disorders – 5th edition, Text Revision (DSM-5 TR) classifications, this syndrome exists not as a distinct class, but rather as an extension of other medical or psychiatric disorders (1). One meta-analysis placed the overall pooled mean prevalence of catatonia across all continents at 9.0% among subjects diagnosed with a range of medical or psychiatric disorders (95% CI = 6.9–11.7, *I*^2^ = 98%) (2). Prevalence estimates of catatonia in children and adolescents are variable at 0.6–17% (3–6). Extant literature on connections between pediatric catatonia with OCD and psychotic disorders is limited to case reports (7, 8). In one study, a catatonic young male demonstrating signs suggesting bipolar disorder began to exhibit a mixture of compulsions, hallucinations, and profound negativism indicating schizophrenia with co-occurring obsessive-compulsive symptoms (9).

Catatonia can appear in the context of psychotic, mood, and obsessive-compulsive disorders. Schizoaffective disorder is characterized by a pattern of concurrent mood and psychotic symptoms with specific timelines. Delusions and hallucinations must be present for two or more weeks in the absence of a major mood episode (i.e., major depressive episodes, manic episodes), but major mood episodes must constitute a majority of the total chronological duration of the illness (1). This disorder can appear similar to other disorders with concurrent mood and psychotic symptoms, such as bipolar I disorder with psychotic features and major depressive disorder with psychotic features. However, the “psychotic features” specifier is incorporated if said symptoms occur exclusively during their respective mood episodes. OCD is characterized by obsessions, which are unwanted and persistent thoughts or urges, and compulsions, which are excessive, repetitive behaviors with a distress reduction component (1).

One difficulty in treating catatonia, particularly pediatric catatonia, is uncovering its associated conditions after mitigating its more severe features. One example is the difficulty of distinguishing a psychotic thought process from an obsessive-compulsive one. Examining an individual’s thought process requires recognizing how a patient interprets topics salient to them. If a patient demonstrates a fear of contamination, this could be interpreted in a few ways. In the absence of core psychotic symptoms such as hallucinations and disorganized speech, a fear of contamination can be construed as delusional if the rationale is implausible and non-amenable to conflicting evidence. Such fear can also be obsessive-compulsive in theme based on how connected the fear is to real events and the degree of conviction to which the patient is convinced that their fear is founded. This interpretation can drastically affect treatment choices after initiation of lorazepam.

Here we present a case of a young girl traumatized by the loss of her mother to suicide who develops signs of an affective psychotic disorder complicated by catatonic features and comorbid obsessive-compulsive symptoms. Table 1 provides a summarized timeline of events.

**Table 1:** Timeline summary of patient events and medications administered over hospital course.

Date	Event	Medication/s	Comment
First hospitalization			
Wk 1	Admission status-post overdose	Restarted FLU 10 mg PO daily Restarted RIS 0.25 mg PO qHS Titrated RIS up to 2 mg PO qHS Started LOR challenge, 1 mg IM Started on LOR 0.5 mg PO TID Started BUP 150 mg PO daily	Collateral information obtained from father Outpatient regimen was reinstated and later up-titrated Patient began decompensating and exhibiting catatonic features that later improved with LOR
Wk 2	Rapid response	Down-titrated LOR to 0.5 mg PO BID Discontinued BUP Titrated LOR back up to 1 mg PO TID	Patient had seizure-like activity and unwitnessed fall. Underwent emergency medicine and neurology workup with negative results LOR re-adjusted and BUP discontinued out of caution Patient’s catatonic features returned
Wk 3	Improvement of symptoms, discharge	Discharge regimen: LOR 1 mg PO TID RIS 2 mg PO qHS	Patient now participating in group activities and tending to ADL’s. Symptoms improved significantly
Second hospitalization			
Wk 4	Decompensation, second admission	LOR titrated to 2 mg PO TID Discontinued RIS Started PAL 234 mg LAI (156 mg on subsequent injections)	Patient presented tearful, anxious, and completely mute. Now exhibiting prominent positive psychotic symptoms Catatonic features returned Medication compliance worsening, now refusing all oral medications Patient given PAL due to history of poor medication compliance and known psychotic symptoms. Medical workup including vitals within normal limits ECT considered, but denied due to external circumstances
Wk 5	Emergence of obsessive-compulsive symptoms	Restarted RIS 1 mg PO qHS Started CYP 2 mg PO TID	Patient alternating between periods of catatonia during the day of intrusive thoughts of nonspecific content during the evening Cyproheptadine started by medicine team to stimulate appetite
Wk 6		Started VAL 250 mg PO BID Started SER 50 mg PO daily Decreased LOR to 1 mg PO TID Decreased CYP to 1 mg PO TID	Patient participating in basic disposition planning when not catatonic, but now also endorsing persecutory delusions and delusions of misidentification Patient now revealing intrusive thoughts and egodystonic obsessions (prolonged handwashing, showering). Also revealing egodystonic thoughts of hurting others Patient now more active on current regimen, managing ADL’s, and interacting appropriately with peers in the unit
Wk 7	Discharge	Discharge regimen: VAL 250 mg PO BID LOR 1 mg PO TID with plans to down-titrate at RTC SER 50 mg PO daily with plans to up-titrate RIS 1 mg PO qHS with plans to discontinue PAL LAI 156 mg as scheduled	Patient discharged to residential treatment center

FLU = fluoxetine, RIS = risperidone, LOR = lorazepam, BUP = bupropion XL, VAL = valproate delayed-release, PAL = paliperidone, LAI = long-acting injectable, CYP = Cyproheptadine, SER = sertraline.

## 2. Case presentation

A 16-year-old girl was brought to the emergency department (ED) after reportedly ingesting an unknown quantity of home medications in a suicide attempt. On presentation to the ED, she could not recount what medications she ingested nor was she willing/able to elaborate on the events leading to this incident. Initial workup during this encounter including metabolic panel, complete blood count (CBC), and urine toxicology was unremarkable. The patient was later medically cleared and transferred to an inpatient psychiatric facility for further treatment/evaluation.

Internal records reviews revealed that the patient had been in and out of psychiatric hospitals for the past 2 years. At one point, she underwent neuropsychological testing to confirm an underlying thought disorder, after which she received referrals to a local First Episode Psychosis Program as well as a residential treatment center. Outpatient providers from other healthcare systems placed her on risperidone and bupropion for unspecified indications during this timeframe. The patient previously presented for suicide attempts similar to the current presentation, with one instance involving a bupropion overdose. Initial impressions during previous hospitalizations included major depressive disorder and post-traumatic stress disorder (PTSD). On one hospitalization approximately 15 months before this encounter, she reportedly exhibited manic behaviors including expansive mood, increasing goal-directed activity, and grandiose ideation, leading to a revised diagnosis of bipolar I disorder with psychotic features.

Collateral information from the father revealed that the patient’s problems began five to six years ago following the unexpected suicide of her mother. He described the patient as lacking drive and struggling to cope with heavy emotions. He reported that the patient had parted ways with her providers at the local First Episode Psychosis Program due to poor follow-up on her part (“we could not get her to buy in,” “she was missing out on sessions”). He also reported that the patient required frequent prompting to comply with her medication regimen with variable success.

On evaluation by the psychiatry team, the patient was a challenging historian who struggled to formulate a linear account of events leading to her admission. She presented with flat affect, concentration difficulties, and a depressed mood. She responded to most inquiries parsimoniously with single-word answers and limited elaboration. The patient initially stated that “nothing was really going on” in the time leading up to her overdose and would respond to further inquiries with variations of “I do not know.” Eventually, she disclosed that she “did not want to be here anymore,” but expressed uncertainty regarding the motivation behind these thoughts. She was restarted on her outpatient regimen of fluoxetine 10 mg PO daily for depression and risperidone 0.25 mg PO qHS for mood stability. Over the first 3 days of her hospitalization, she appeared increasingly withdrawn. The patient did not engage with peers or groups in the inpatient milieu and often sat in the activity room engaging in minimal to no recreational activity. The patient’s risperidone was subsequently titrated up to 2 mg PO qHS to limited effect.

On the fourth day of her hospitalization, the patient became mute and non-responsive. She stopped eating and drinking. She began exhibiting posturing when prompted to drink water such that she would hold a cup in a prolonged fixed position without bringing it to her mouth. A lorazepam challenge [1 mg intramuscularly (IM)] was administered with partial response. Following partial response, the patient was started on scheduled lorazepam at 1 mg PO TID. The patient appeared to improve on oral lorazepam such that she began responding minimally, eating with prompting, and making basic needs known. She began disclosing symptoms of severe depression, lack of energy, lack of motivation, and later thoughts of suicide. At this point, the patient was taken off fluoxetine and started on bupropion 150 mg PO daily for norepinephrine effects.

Over the course of her hospitalization, the patient appeared to improve such that she ate, drank, and participated in unit activities. She reported feelings of sedation and tiredness which led the team to down-titrate her scheduled lorazepam to 0.5 mg PO BID. On the tenth day of her hospitalization, a rapid response alert was called and staff arrived to find her exhibiting seizure-like activity, but maintaining awareness of her environment. The patient underwent evaluations by ED and Neurology services. Bupropion was discontinued as a precaution. Her systemic vital signs were stable. Initial workup including magnetic resonance imaging (MRI), electroencephalogram (EEG), CBC, metabolic panel (including liver function tests, electrolytes, renal function, urine toxicology), and heavy metal study were all unremarkable. Following this event, the patient’s catatonic features returned including echopraxia, mutism, and negativism. Her lorazepam schedule was titrated back to 1 mg PO TID such that these features began to remit over 8 days. Upon observation that the patient stabilized such that she participated in unit activities and tended to her activities of daily living (ADL’s), the team prepared the patient for discharge with outpatient follow-up.

Three days following discharge, the patient returned to the hospital in a worse condition than her first admission. She was tearful, anxious, and completely mute. She stopped eating and drinking and now engaged in only limited purposeful movement. When she returned home from her recent hospitalization, the patient’s father described her as acting “odd” but overall improved. This “odd” behavior progressed when the patient’s father and godmother arranged a group dinner. Per the father’s report, the patient spent a significant amount of time with this godmother before her mother’s suicide. During dinner, the godmother noticed that the patient was making “bizarre” statements but was otherwise “able to talk about how she is feeling.” At home that evening, the patient began exhibiting profoundly paranoid behavior. The patient was pacing the hallways asking about the computer, the microwave, and other electronics. She commented that these devices were talking to her. The father offered her food to which she began expressing suspicion and later refusal. He reported that the patient was compliant with medications and that he oversaw her regimen. However, this was difficult to verify.

The patient’s catatonic features continued to relapse and remit over several weeks during this hospitalization. She would approach the inpatient nursing station in distress and occasionally attempt to push on closed doors randomly without clear motive. Unit staff reportedly observed patient responding to internal stimuli. She required aspiration precautions as she was failing to swallow her saliva. Electroconvulsive therapy (ECT) was considered but rendered a non-option due to institutional policies barring the administration of ECT for minors and a lack of qualified facilities in the region that offered ECT for minors. The patient was placed on scheduled lorazepam titrated to 2 mg PO TID with intramuscular lorazepam PRN if she either refused or failed to tolerate oral medications. Due to the patient’s history of poor medication compliance and known history of psychotic symptoms, she was also started on long-acting injectable paliperidone at 234 mg (156 mg on subsequent injections).

On the fourth day of this lorazepam course, the patient’s behaviors took on a dimension not seen in previous hospitalizations. The team observed that the patient spent prolonged periods washing her hands and taking showers. She requested that staff wear and change their gloves before interacting with her and asked for food containers to be opened in front of her before consumption. She appeared to alternate between periods of catatonia during the day and obsessive and intrusive thoughts during the evening. She began disclosing feelings that the environment around her was unclean and that she was experiencing ego-dystonic thoughts of harming others. She reported feeling pregnant (note: Urine hCG was negative) and expressed concerns that staff were attempting to harm her. During periods of remission, she tended to basic ADL’s. She continued to demonstrate paranoia, though whether this was psychotic or obsessive-compulsive in nature was unclear. The patient’s oral risperidone was adjusted throughout her course while her paliperidone reached steady state. Her catatonic symptoms continued to wax and wane, prompting initiation of delayed-release valproate 250 mg PO BID for augmentation.

On this regimen, the patient’s catatonic features went into complete remission over several weeks. She continued to exhibit intrusive thoughts and at times persecutory delusions such as fears that the hospital was “doxxing” her and that her intrusive thoughts resulted from a tumor. Sertraline 50 mg PO daily was added to her regimen to address her obsessive-compulsive symptoms with plans to titrate upwards in outpatient. By the 50th day of her hospitalization, the patient began playing cards with her peers and interacting during group sessions. Her delusions and obsessions continued to persist, albeit less pronounced compared to initial admission. The patient was eventually discharged to a residential treatment center.

## 3. Discussion and conclusions

In the presented case, a sixteen-year-old female experienced the traumatic loss of her mother to suicide after which she developed mood symptoms that culminated in multiple suicide attempts. During her hospitalization, the patient’s course was complicated by the emergence of catatonic features that responded to benzodiazepines with increasing variability. As her catatonia relapsed and remitted during her hospital course, she began demonstrating a pattern of comorbid symptoms that appeared to overlap across multiple disorders: 1) severe psychomotor retardation, anhedonia, low energy, and concentration difficulties as seen in depressive disorders, 2) negativism and thought disorganization as seen in primary psychotic disorders, and 3) severe anxiety as the result of intrusive thoughts and obsessions with contamination coupled with time-consuming compulsions as seen in obsessive-compulsive disorders. This pattern was further complicated by reported mania as well as unexplored posttraumatic sequela in the aftermath of the patient’s mother’s death.

Of relevance to this case is the constellation of comorbidities that may have contributed to the patient’s catatonia. Catatonia is recognized as occurring primarily in the context of other disorders. However, its pathogenesis relative to other disorders is not fully understood (1). One proposed explanation is that catatonia is primarily a motor disorder with Parkinsonian features built on Kahlbaum’s original conception of the phenomenon (10, 11). The motor behaviors seen in this syndrome have been suggested to be a dysfunction of frontal lobe basal ganglia circuitry with implicated neurotransmitters including GABA, glutamate, and dopamine (12–14). Other proponents described catatonia as a derangement of the evolutionarily ingrained fear response (15, 16). In this explanation, the fear response activates when an external threat can be localized. However, this fear response transforms into an anxiety response when the associated sense of imminent doom continues even in the absence of an external threat. The varying behavioral characteristics of catatonia, ranging from catalepsy to excitement, are posited to be aberrations of the fear response as it transitions into the anxiety response. For example, the stupor seen in catatonia is viewed as the anxiety-related aberration of *tonic immobility*, an animal defense strategy and fear response characterized by muscle tension. This view may at least partially explain the efficacy of benzodiazepines in alleviating catatonic symptoms. Here we have a case of a pediatric patient with catatonia whose associated symptoms and responses to treatment appear to fall somewhere between these theoretical frameworks.

As demonstrated in this patient, teasing apart concurrent comorbidities can be diagnostically challenging. The salient challenge here involved identifying the obsessive-compulsive features in this patient. For much of her early course, the patient exhibited positive psychotic symptoms, including ideas of reference and paranoia, and progressively worsening negativism, including alogia, avolition, and diminished emotional expression. These symptoms were precipitated by reports of manic and major depressive episodes in the time leading up to the presenting study. During her course, she self-described the presence of “intrusive thoughts” relating to contamination. These thoughts at first appeared delusional. However, she demonstrated both insight into the irrationality behind these thoughts and the extent to which these thoughts contributed to her ongoing distress. Concern for concurrent OCD emerged during her second hospitalization when the patient began engaging in repetitive and time-consuming behaviors, including handwashing and prolonged showering. There were reports of the patient responding to internal stimuli, which would normally preclude the diagnosis of OCD. However, further inquiries by the authors revealed that during these episodes, the patient was engaging in protracted preoccupation with her contamination fears that appeared more consistent with internal rumination. The patient expressed suspicion towards others in a manner that could be construed as paranoia in the context of psychosis. However, re-evaluation of these behaviors suggested that this paranoia may have been an extension of her worry regarding her contamination fears. This discrepancy does not preclude the presence of a primary psychotic disorder, but rather, it demonstrates the result of two disorders with intermingling symptoms that can be construed for one another. This relationship was complicated further by the patient’s catatonia.

Treatment of catatonia should take precedence in such a presentation. Left untreated, patients with catatonia may develop rhabdomyolysis, malnutrition, dehydration, and in some cases development of deep vein thrombosis or pulmonary embolism (17–20). Malignant catatonia is a rare but lethal variant of this syndrome characterized by autonomic instability, fever, and accompanying lab aberrancies such as elevated CPK, low serum iron, and leukocytosis (21). First-line management involves trialing the patient on lorazepam at a dose of 1–2 mg, preferably by intravenous route for response time with close monitoring for adverse effects over 3 hours. Positive treatment response can be confirmed by comparing scores for the Bush-Francis Catatonia Rating Scale before and after treatment administration (13, 22). Positive treatment response is indicated by a 50% decrease between pre-and post-scores, however negative response does not exclude the diagnosis of catatonia. Administrations can be repeated up to 20 mg without sedation as some patients can demonstrate GABA dysregulation or benzodiazepine tolerance. Second-line management in the event of benzodiazepine treatment failure involves using ECT (if available), which has demonstrated response rates of up to 80–100% among child and adolescent populations with catatonia (23). If ECT is not feasible, alternative oral medications such as valproate and zolpidem can be used as augmenting agents. If the presence of OCD can be confirmed, a selective serotonin reuptake inhibitor such as sertraline may be appropriate. Of note, the authors acknowledge the use of bupropion which is not approved for pediatric use. The decision to initiate this medication was due to the failure of multiple interventions (i.e., sertraline and fluoxetine), attempts by the team to introduce norepinephrine effects to manage depressive symptoms, and because records reviews revealed that the patient previously benefited from this medication.

This case report affirms the importance of addressing associated psychiatric or medical conditions in addition to treating catatonia. The distinguishing feature of this case report is the challenge of identifying said conditions and their association with catatonia. One limitation to this case report was the limited data available to explore the contribution of the patient’s complex trauma history to her circumstance. Institutional delays in the release of outpatient records characterizing the patient’s trauma history prevented their inclusion in the case presentation. Adverse childhood experiences such as deprivation, abuse, or trauma, may precipitate the onset of catatonia (24). Reports of pediatric catatonia associated with posttraumatic stress disorder are limited to case reports (25, 26). However, their presence in the literature only highlights the need for further studies investigating the underlying causes of catatonia, not just in obsessive-compulsive disorders but across other conditions as well.

## Data availability statement

The original contributions presented in the study are included in the article/supplementary material, further inquiries can be directed to the corresponding authors.

## Ethics statement

Written informed consent was obtained from the individual(s), and minor(s)’ legal guardian/next of kin, for the publication of any potentially identifiable images or data included in this article.

## Author contributions

WT and CY wrote the manuscript, organized discussions, compiled relevant records for review, and revised the manuscript where directed. TV-H was the primary resident clinician and was involved in discussions, manuscript revision, and approval of the final draft. MB was part of the care team and was involved in discussion of the patient’s case. CF was the supervising attending physician on the case. All authors contributed to the article and approved the submitted version.
